# Supplementary material for: MetaQTL: a package of new computational methods for the meta-analysis of QTL mapping experiments
Source: BMC Bioinformatics. 2007 Feb 8;8:49. doi: 10.1186/1471-2105-8-49 (PMC1808479; doi:10.1186/1471-2105-8-49)
Supplement: Additional File 7 — MetaQTL Package : jar file and tutorial. This Zip archive contains both the MetaQTL JAR file and the files of the tutorial. [file 1471-2105-8-49-S7.zip › org.inra.metaqtl/doc/index-files/index-15.html]

P-Index


|  |  |  |
| --- | --- | --- |
| **Overview** \| Package \| Class \| Use \| **Tree** \| **Deprecated** \| **Index** \| **Help** | |  |
| **PREV LETTER**   **NEXT LETTER** | **FRAMES**    **NO FRAMES**     **All Classes** |  |


A B C D E F G H I K L M N O P Q R S T U V W X Y Z 

---


## **P**

**PaletteColor** - Class in org.metaqtl.graph: **PaletteColor()** - Constructor for class org.metaqtl.graph.PaletteColor: **parent** - Variable in class org.metaqtl.TreeNode: The parent of the node **parents** - Variable in class org.thalia.bio.entity.Population: **parse(String[])** - Method in class org.metaqtl.main.CmdLineParser: Extract the options and non-option arguments from the given list of command-line arguments. **parse(String[], Locale)** - Method in class org.metaqtl.main.CmdLineParser: Extract the options and non-option arguments from the given list of command-line arguments. **parseChromList(String)** - Static method in class org.metaqtl.main.MetaMain: **parseCmdLine(String[])** - Method in class org.metaqtl.main.MetaMain: **parseCross(String)** - Static method in class org.thalia.bio.util.BioCrossTypes: Parse the given string and return if valid the type of cross and the number of generations. **parseDouble(String)** - Static method in class org.thalia.bio.util.NumberFormat: This method parse the given string to a double value. **parseType(String)** - Static method in class org.thalia.bio.util.BioLocusTypes: **pi** - Variable in class org.metaqtl.EMResult: The mixing proportions. **pnames** - Variable in class org.metaqtl.QtlPartition: The partition names. **Population** - Class in org.thalia.bio.entity: Class Description Here **Population()** - Constructor for class org.thalia.bio.entity.Population: **Population(String, IBioEntity)** - Constructor for class org.thalia.bio.entity.Population: **POPULATION** - Static variable in interface org.thalia.bio.IBioEntityTypes: The integer code for Population entity **POS\_COMMON** - Static variable in class org.metaqtl.graph.ChromLayer: **POS\_COMMON\_COLOR** - Static variable in class org.metaqtl.graph.MetaGraphPar: **position** - Variable in class org.metaqtl.Qtl: The QTL most probable location on the chromosome. **position** - Variable in class org.thalia.bio.entity.bean.LocusBean: **printHelp()** - Method in class org.metaqtl.main.A2Xml: **printHelp()** - Method in class org.metaqtl.main.ConsMap: **printHelp()** - Method in class org.metaqtl.main.InfoMap: **printHelp()** - Method in class org.metaqtl.main.MapView: **printHelp()** - Method in class org.metaqtl.main.MetaDB: **printHelp()** - Method in class org.metaqtl.main.MetaMain: **printHelp()** - Method in class org.metaqtl.main.MMapView: **printHelp()** - Method in class org.metaqtl.main.MQTLView: **printHelp()** - Method in class org.metaqtl.main.QTLClust: **printHelp()** - Method in class org.metaqtl.main.QTLClustInfo: **printHelp()** - Method in class org.metaqtl.main.QTLModel: **printHelp()** - Method in class org.metaqtl.main.QTLProj: **printHelp()** - Method in class org.metaqtl.main.QTLTree: **printHelp()** - Method in class org.metaqtl.main.Xml2A: **printHelp()** - Method in class org.thalia.metaqtl.main.MetaCMS: **printHelp()** - Method in class org.thalia.metaqtl.main.MetaDraw: **printHelp()** - Method in class org.thalia.metaqtl.main.MetaMapDraw: **printHelp()** - Method in class org.thalia.metaqtl.main.MetaSimu: **printHelp()** - Method in class org.thalia.metaqtl.main.MetaSimuStat: **printImgFormat(int)** - Static method in class org.metaqtl.main.MetaMain: **printLicense(String, String)** - Static method in class org.metaqtl.main.MetaMain: **printUsage()** - Method in class org.metaqtl.main.A2Xml: **printUsage()** - Method in class org.metaqtl.main.ConsMap: **printUsage()** - Method in class org.metaqtl.main.InfoMap: **printUsage()** - Method in class org.metaqtl.main.MapView: **printUsage()** - Method in class org.metaqtl.main.MetaDB: **printUsage()** - Method in class org.metaqtl.main.MetaMain: **printUsage()** - Method in class org.metaqtl.main.MMapView: **printUsage()** - Method in class org.metaqtl.main.MQTLView: **printUsage()** - Method in class org.metaqtl.main.QTLClust: **printUsage()** - Method in class org.metaqtl.main.QTLClustInfo: **printUsage()** - Method in class org.metaqtl.main.QTLModel: **printUsage()** - Method in class org.metaqtl.main.QTLProj: **printUsage()** - Method in class org.metaqtl.main.QTLTree: **printUsage()** - Method in class org.metaqtl.main.Xml2A: **printUsage()** - Method in class org.thalia.metaqtl.main.MetaCMS: **printUsage()** - Method in class org.thalia.metaqtl.main.MetaDraw: **printUsage()** - Method in class org.thalia.metaqtl.main.MetaMapDraw: **printUsage()** - Method in class org.thalia.metaqtl.main.MetaSimu: **printUsage()** - Method in class org.thalia.metaqtl.main.MetaSimuStat: **proba** - Variable in class org.metaqtl.Chromosome: Probabilities defined along the chromosome. **proba** - Variable in class org.metaqtl.QtlPartition: The probabilities of each Qtl to belong to each partition. **PROBA\_BIN** - Static variable in class org.metaqtl.graph.MetaGraphPar: **PROBA\_FROM\_COLOR** - Static variable in class org.metaqtl.graph.MetaGraphPar: **PROBA\_PALETTE** - Static variable in class org.metaqtl.graph.MetaGraphPar: **PROBA\_TO\_COLOR** - Static variable in class org.metaqtl.graph.MetaGraphPar: **proj** - Variable in class org.metaqtl.Qtl: The QTL projection properties. **properties** - Variable in class org.thalia.bio.entity.bean.BioEntityBean: **PropertyBean** - Class in org.thalia.bio.entity.bean: Class Description Here **PropertyBean()** - Constructor for class org.thalia.bio.entity.bean.PropertyBean: **PropertyBean(String, String)** - Constructor for class org.thalia.bio.entity.bean.PropertyBean: **PVALUE** - Static variable in interface org.metaqtl.IBioLGroupProperties: The key for the p-value. **pvalue** - Variable in class org.metaqtl.MetaChrom: The p-value of this chromosome. **pvalue** - Variable in class org.metaqtl.MultiFitResult: The p-value of the model. **PYTHAG(double, double)** - Static method in class org.metaqtl.numrec.NumericalUtilities: Computes sqrt(a^2+b^2)

---


|  |  |  |
| --- | --- | --- |
| **Overview** \| Package \| Class \| Use \| **Tree** \| **Deprecated** \| **Index** \| **Help** | |  |
| **PREV LETTER**   **NEXT LETTER** | **FRAMES**    **NO FRAMES**     **All Classes** |  |


A B C D E F G H I K L M N O P Q R S T U V W X Y Z 

---
